# Atypical Fungal Rash

**DOI:** 10.5811/cpcem.2019.1.41329

**Published:** 2019-02-26

**Authors:** Fadi W.M. Adel, Venkatesh Bellamkonda

**Affiliations:** * Mayo Clinic, Department of Internal Medicine, Rochester, Minnesota; † Mayo Clinic, Department of Emergency Medicine, Rochester, Minnesota

## CASE PRESENTATION

A 73-year-old man with rheumatoid arthritis on prednisone (10 milligrams [mg] daily routinely, and increased to 40 mg daily during frequent exacerbations) presented to the emergency department with chills and a leg rash. Two weeks prior, he noticed redness on his right thigh, with black spots developing later. His vital signs were normal, and his physical examination was significant for a 6 × 10 centimeter (cm) red, warm patch with 0.5 cm indurated black papules and ulcers (Image). His lab work-up was unremarkable. Periodic acid–Schiff–diastase and Gram stains of a punch biopsy sample of one papule demonstrated variably sized yeast and hyphal fungal elements. *Purpureocillium lilacinum* grew, thus clinching the diagnosis.

## DISCUSSION

Although often dismissed as a contaminant, *P. lilacinum* is an emerging fungal agent implicated in cutaneous and pulmonary diseases in immunocompetent and, more often, immunocompromised hosts. The fungus commonly grows on decaying organic material in soil.^1^ We are aware of very few cases of cutaneous infection by this fungus reported in the literature and, to our knowledge, these are the only images with black papules. According to Saghrouni et al., successful cure has been achieved with griseofulvin, itraconazole, ketoconazole, and voriconazole in other cases.^2^,^3^ Voriconazole was initiated for this patient; however, he was lost to follow-up. This vignette demonstrates the importance of recognizing uncommon fungal infections, especially in immunocompromised patients.

**CPC-EM Capsule**

What do we already know about this clinical entity?

*Purpureocillium lilacinum is a filamentous, soil-dwelling fungus and an emergent pathogen implicated in eye and skin infections, nasal septal perforations, and lung cavitation*.

What is the major impact of the image(s)?

*This is the first report of cutaneous P. lilacinum presenting as black papular lesions on an erythematous base. Previously, it was isolated from abscesses and tattoo-related red papules*.

How might this improve emergency medicine practice?

*This image will aid emergency providers to recognize similar-appearing lesions as potential cutaneous mycosis, and subsequently guide appropriate management and/or referral*.

## Figures and Tables

**Image:**
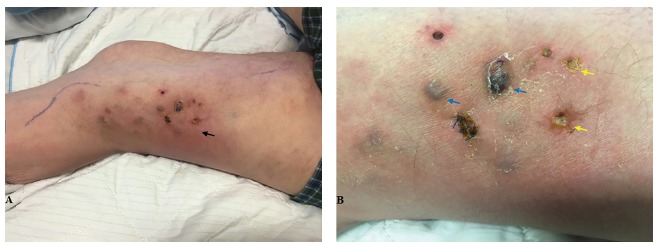
*Purpureocillium lilacinum* in an immunocompromised host. A) The erythematous, warm base (black arrow) is on the medial surface of the patient’s thigh. B) A close-up view of the lesion demonstrates black papules (blue arrows) and ulcers (yellow arrows).
